# Effect of Zinc Supplementation on Glycemic Control in Newly Diagnosed Patients With Type 2 Diabetes Mellitus

**DOI:** 10.7759/cureus.69180

**Published:** 2024-09-11

**Authors:** Gurpreet S Chhina, Ajay Chhabra, Sumedh R Luthra, Saloni Khattar, Priyanka Singh, Shivansh Luthra

**Affiliations:** 1 Department of Medicine, Government Medical College Amritsar, Amritsar, IND

**Keywords:** diabetes mellitus, glycemic control, insulin resistance, type 2 diabetes, zinc supplementation

## Abstract

Introduction: Diabetes mellitus (DM) is a spectrum of metabolic disorders primarily characterized by elevated blood glucose levels. Type 2 DM (T2DM), the most common form, often requires adjunctive therapies to improve glycemic control and mitigate associated risks. Zinc has been implicated in glucose metabolism and insulin function, prompting this study to evaluate the impact of zinc supplementation on glycemic control in newly diagnosed T2DM patients.

Methods: A randomized, placebo-controlled trial was conducted involving 80 newly diagnosed T2DM patients. Participants were randomly assigned to receive either zinc supplementation (50 mg/day) or a placebo, in conjunction with standard oral hypoglycemic medication, metformin. Key indicators, including fasting blood glucose (FBG), postprandial blood glucose (PPBG), hemoglobin A1c (HbA1c), and lipid profiles, were measured at baseline and after the 12-month intervention period.

Results: The group receiving zinc supplementation demonstrated significant reductions in FBG, PPBG, and HbA1c levels compared to the placebo group. The mean FBG in the intervention group decreased by 21.52 mg/dL, PPBG decreased by 47.53 mg/dL, and HbA1c decreased by 0.79%. Additionally, zinc supplementation led to notable decreases in low-density lipoprotein (LDL) cholesterol by 25.06 mg/dL, triglycerides by 22.2 mg/dL, and total cholesterol levels by 26.67 mg/dL. However, no significant changes were observed in high-density lipoprotein (HDL) cholesterol, very-low-density lipoprotein (VLDL) cholesterol, or erythrocyte sedimentation rate (ESR).

Discussion: The findings suggest that zinc supplementation may be a beneficial adjunctive therapy in the early management of T2DM, contributing to improved glycemic control and favorable changes in lipid profiles. However, its effect on HDL, VLDL, and ESR was insignificant, indicating the need for further research to better understand the broader implications of zinc in T2DM management.

## Introduction

A variety of metabolic diseases characterized by high blood glucose levels are collectively referred to as diabetes mellitus (DM) [1]. Diabetes is a chronic illness characterized by abnormalities in the action or secretion of insulin, or sometimes both [2,3]. The pancreas secretes insulin, a hormone that plays a crucial role in transferring glucose from the bloodstream into the body's cells, where it can be used as fuel [4]. Diabetes affects various organs, including the eyes, nerves, and kidneys [5].

About 90% of diabetes cases are type 2 diabetes, which is characterized by decreased insulin levels, β-cell dysfunction, insulin resistance, and the eventual loss of β-cells [6]. Insulin, stored in pancreatic β-cells as a hexamer containing two zinc ions, is released during β-cell degranulation [7]. Zinc ions co-secreted with insulin play a crucial role in insulin production and function by preventing monomeric insulin from becoming amyloidogenic [8,9].

Clinical investigations into the effect of zinc supplementation on glycemic control in individuals with type 2 diabetes have yielded inconsistent findings. Some trials have reported benefits, such as improved glycemic control, reduced insulin resistance, and decreased levels of inflammatory markers [10]. However, other investigations have found no discernible impact of zinc supplementation on glycemic indices or insulin sensitivity [11]. These contradictory results may be due to various factors, including differences in research populations, baseline zinc levels, supplementation dosage and duration, and concomitant treatments or medications [12,13]. Additionally, most studies have focused on patients with established T2DM, with few examining the potential significance of zinc supplementation in the early stages of the disease.

The current study set out to investigate the effects of zinc supplementation on the glycemic control of newly diagnosed type 2 diabetes patients. The goal was to determine whether zinc supplementation could serve as an adjunctive treatment in the early stages of type 2 diabetes care, a period when glycemic control is crucial for preventing or delaying complications. This was achieved by focusing on patients who were still in the early stages of the disease.

## Materials and methods

The study was conducted in the Medicine Outpatient Department (OPD) of Guru Nanak Dev Hospital (GNDH), Government Medical College, Amritsar. The population frame included individuals newly diagnosed with Type 2 DM (T2DM) based on the American Diabetes Association (ADA) standards. Over a 12-month period (2023-2024), all adult patients presenting to the OPD with symptoms such as easy fatigability, polyuria, polyphagia, polydipsia, or recognized diabetes risk factors were assessed for the research. Patients referred from other facilities after an initial urine glucose test, as well as those from other associated departments, were included. Diabetes screening was conducted using hemoglobin A1c (HbA1c) and fasting blood glucose (FBG) assays. Patients who satisfied the eligibility requirements were enrolled in the trial.

Study design

This study was designed as an open-label, prospective, randomized trial.

Inclusion criteria

The inclusion criteria included newly diagnosed Type 2 diabetes patients without complications, who visited the Medicine OPD at GNDH and were 20 years of age or older. Both male and female patients participated in the study.

Exclusion criteria

Patients with Type 1 diabetes, Type 2 diabetes with complications, diabetes during pregnancy, chronic kidney disease, chronic liver disease, chronic pancreatitis, or inflammatory bowel disease (IBD) were excluded from the research. The study also excluded patients already taking zinc supplements, immunomodulatory medications, or chelating agents, as well as those on hemodialysis and those with pre-diabetes or an HbA1c score greater than 9.5%.

Consent

All patients enrolled in the study provided informed consent using a form prepared in the vernacular language. The Institutional Ethical Committee of Government Medical College, Amritsar, approved this form to ensure that ethical standards for patient involvement were met.

The research population included new patients who visited the OPD with risk factors for DM, those referred from other institutes, and those admitted to GNDH after testing positive for blood glucose or being referred from other departments. After initial selection, informed consent was obtained for participation in the trial, followed by the necessary examinations. Patients were then tested for FBG and hemoglobin A1c (HbA1c). After excluding patients with impaired fasting glucose (IFG), impaired glucose tolerance (IGT), or normal glucose readings, 80 individuals were deemed suitable for further evaluation.

The remaining patients underwent abdominal ultrasonography to rule out chronic renal and liver disease. A fundus examination was performed to rule out diabetic retinopathy, along with an ECG, blood urea, serum creatinine, and a standard urine test. After completing these examinations, the 80 eligible patients were randomly assigned to two groups using computer-generated random numbers. Group A and Group B consisted of 40 patients each.

Patients included in the trial were explicitly instructed to continue their normal daily routines and to avoid taking any zinc supplementation other than what was prescribed as part of the study protocol. Additionally, patients were instructed to fast for at least eight hours before reporting to the OPD for their investigations.

Investigation profile

Comprehensive data collection included a detailed history, vital signs, height, weight, and clinical examination of all patients, systematically recorded in a proforma. Body mass index (BMI) was calculated for each participant. The proforma spreadsheet documented investigation results, including HbA1c, FBG, postprandial blood glucose (PPBG), renal function tests, fasting lipid profile, and full hemogram. Blood samples were collected under fasting conditions (8 mL) and in the postprandial state (3 mL). These samples were stored in a cold room until analysis.

Intervention

Participants in Group 2 received an additional daily dose of 50 mg of zinc tablets along with their standard oral hypoglycemic medications, while Group 1 received only the standard oral hypoglycemic drugs available at the hospital. All subjects were instructed to maintain their usual activities and avoid taking any additional vitamin supplements. They were also advised to visit every three months for medication refills and to report any adverse effects. Zinc dosage was selected based on a literature review on safety, with a dosage of 50 mg/day chosen for this study. After one year, measurements including BMI, FBG, PPBG, HbA1c, lipid profile, serum zinc levels, CBC, urine analysis, erythrocyte sedimentation rate (ESR), serum urea, and serum creatinine were recorded and compiled into a master chart. Follow-up was conducted at 1, 3, and 6 months, and after completion of treatment, patients were also called back to check for zinc toxicity or any other adverse effects.

Glucose and zinc test principle

The glucose test was conducted using the Cobas C311 enzymatic method with hexokinase. HbA1c levels were determined using the turbidimetric inhibition immunoassay (TINIA) method for hemolyzed whole blood. Serum zinc concentrations were automatically calculated by the analyzer using a calibration curve and reported in micrograms per deciliter (µg/dL).

Statistics

The statistical approach for this study was carefully planned to ensure the validity and reliability of the findings. Data analysis was conducted using IBM SPSS Statistics for Windows, Version 29 (Released 2023; IBM Corp., Armonk, New York), with continuous variables compared using paired and unpaired t-tests, and categorical variables assessed through chi-square and Fisher's exact tests. Statistical significance was defined as p < 0.05.

Sample size calculation

For a 10% difference in HbA1c, assuming a standard deviation of 0.159 based on prior studies, a significance level of 0.05, and a power of 80%, the required sample size per group was calculated using the formula: 

$n = \frac{2 \sigma^2 (Z_{\alpha/2} + Z_{\beta})^2}{\delta^2}$

where σ is the standard deviation (0.159), Zα/2​ is 1.96 for a 95% confidence level, ZβZ is 0.84 for 80% power, and δ is the expected difference in means (0.10). The calculation determined that a sample size of 40 per group is required.

## Results

Out of the 323 individuals assessed for eligibility, 203 were excluded, including 170 for not meeting the inclusion criteria and 33 who declined to participate. This left 120 participants who were randomized into the study. In the allocation phase, 65 participants were assigned to the placebo group, with 55 receiving the intervention and 15 unable to tolerate metformin. In the zinc group, 55 participants were allocated, with 50 receiving the intervention and 5 unable to tolerate metformin. During the follow-up period, 15 participants from the placebo group and 10 from the zinc group were lost to follow-up. Ultimately, 40 participants from each group were analyzed for the study.

**Figure 1:**
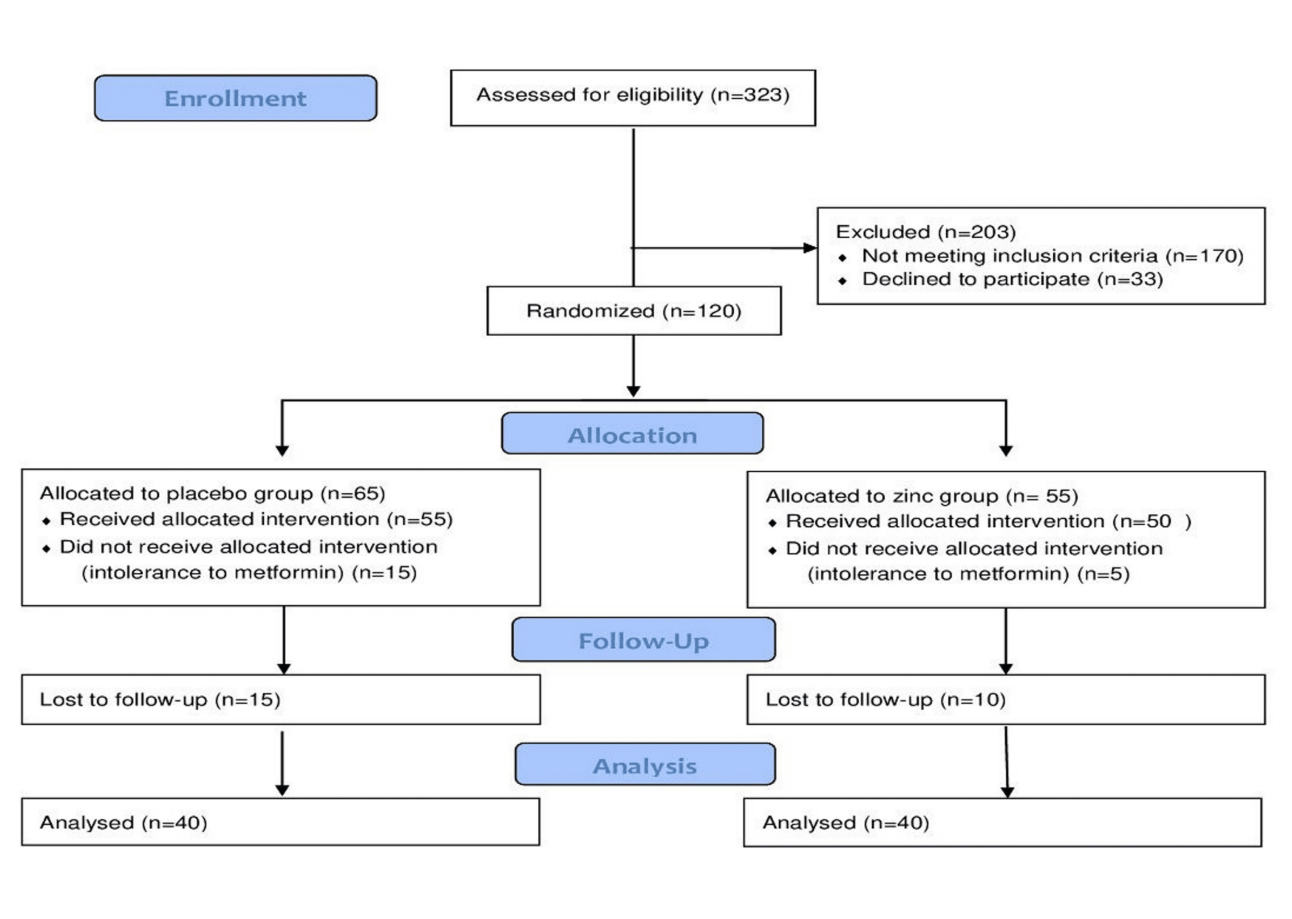
CONSORT (Consolidated Standards of Reporting Trials) diagram for the sample size

Patients who met the inclusion criteria were randomized into two groups: Group A and Group B, each consisting of 40 participants. The randomization was carried out using computer-generated random numbers to ensure equal distribution between the two groups. Allocation concealment was maintained using sequentially numbered, opaque, sealed envelopes (SNOSE) that were prepared in advance. After obtaining consent and completing the initial screening, a designated research staff member, who was not involved in the clinical assessment, opened the next envelope in sequence to determine group assignment. This process ensured that the allocation remained concealed from both the enrolling investigators and participants until the moment of assignment, minimizing selection bias. The sample size was 40 participants per group, as described in the CONSORT (Consolidated Standards of Reporting Trials) diagram (Figure 1).

After meeting the study's qualifying criteria, 80 patients were included and divided into two groups of 40 each. These patients were monitored for approximately one year (Table 1).

**Table 1:** Baseline variables of the treatment groups BMI: body mass index, FBG: fasting blood glucose, PPBG: postprandial blood glucose, HbA1c: glycated hemoglobin, LDL: low-density lipoprotein, PRE: pre-intervention.

Variable	Group 1 (Placebo)	Group 2 (Zinc)	p-value
Age (years)	48.55±8.67	47.62±7.49	0.611
BMI (kg/m²) - PRE	27.99±3.22	27.60±4.06	0.636
Sex (female/male)	23/17	21/19	0.653
FBG (mg/dL) - PRE	159.60±21.57	152.12±22.23	0.132
PPBG (mg/dL) - PRE	254.35±44.14	257.80±65.27	0.783
HbA1c (%) - PRE	7.57±0.69	7.68±0.65	0.488
LDL (mg/dL) - PRE	95.67±17.18	80.62±12.58	0.001
Serum zinc (µg/dL) - PRE	70.17±14.25	67.32±8.11	0.275

Data collected and analyzed included age, gender, BMI, FBG, postprandial blood glucose (PPBG), hemoglobin A1c (HbA1c), low-density lipoprotein (LDL) cholesterol, triglycerides, high-density lipoprotein (HDL) cholesterol, total cholesterol, very-low-density lipoprotein (VLDL) cholesterol, ESR, and serum zinc levels. The study findings are as follows: Over the one-year monitoring period, the 80 patients were divided into two groups: Group 1 received placebo supplementation plus metformin, while Group 2 received zinc supplementation plus metformin, as shown in Table 2. 

**Table 2:** Overview of age and BMI The age group is subdivided into ranges, and their respective counts and percentages are listed for both Group A and Group B. BMI (pre-intervention) and BMI (post-intervention) columns represent the pre- and post-treatment BMI values for both Group A and Group B. Age-wise distribution: p-value 0.611, No significant difference in age groups between both the groups. Pre-intervention BMI: p-value: 0.636, no significant difference in BMI distribution before the intervention. Post-intervention BMI: p-value: 0.756, no significant difference in BMI distribution after the intervention. BMI: body mass index, PRE: pre-treatment, POST: post-intervention.

Category	Subcategory	Group A - No. (%)	Group B - No. (%)	p-value
Age group	20-30	2 (5.00)	1 (2.50)	
	31-40	5 (12.50)	5 (12.50)	
	41-50	17 (42.50)	22 (55.00)	
	51-60	14 (35.00)	9 (22.50)	
	61-70	2 (5.00)	3 (7.50)	
Total		40 (100.00)	40 (100.00)	
Mean age (years)		48.55±8.67	47.62±7.49	0.611
BMI (PRE)	<18.5 (Underweight)	0 (0.00)	0 (0.00)	
	18.5-24.9 (Normal)	8 (20.00)	8 (20.00)	
	25.0-29.9 (Overweight)	23 (57.50)	24 (60.00)	
	>30.0 (Obese)	9 (22.50)	8 (20.00)	
Total		40 (100.00)	40 (100.00)	
Mean BMI (kg/m²)		27.99±3.22	27.60±4.06	0.636
BMI (POST)	<18.5 (Underweight)	0 (0.00)	0 (0.00)	
	18.5-24.9 (Normal)	8 (20.00)	8 (20.00)	
	25.0-29.9 (Overweight)	21 (52.50)	23 (57.50)	
	>30.0 (Obese)	11 (27.50)	9 (22.50)	
Total		40 (100.00)	40 (100.00)	
Mean BMI (kg/m²)		28.05±3.23	27.80±3.87	0.756

Observations and comparisons were made across several parameters, including age, gender, BMI, FBG, postprandial blood glucose (PPBG), hemoglobin A1c (HbA1c), lipid profiles (LDL cholesterol, triglycerides (TG), HDL cholesterol, total cholesterol (CHL), and VLDL cholesterol), ESR, and serum zinc levels. The age-wise distribution of the patients is shown in Table 1.

According to the chi-square test, the gender distribution between the two groups yielded a p-value of 0.653, indicating no statistically significant difference. In the study, the distribution of sex among participants was examined across the two groups. In Group A, there were 23 females, constituting 57.5% of the group, and 17 males, making up 42.5%. Conversely, Group B comprised 21 females (52.5%) and 19 males (47.5%). The total number of participants in each group was 40. Statistical analysis of the sex distribution using a chi-square test revealed a value of X² = 0.202 with 1 degree of freedom and a p-value of 0.653, indicating no significant difference in sex distribution between the two groups. Similarly, the lack of statistical significance for both age and gender suggests no differences between the groups, meaning that both groups consist of individuals with comparable baseline demographic characteristics.

Before the intervention, both Group A and Group B exhibited similar BMI distributions. In Group A, none of the participants were underweight; 8 participants (20%) were of normal weight, 23 participants (57.5%) were overweight, and 9 participants (22.5%) were obese. The mean BMI for Group A was 27.99 ± 3.22 kg/m². Group B showed a similar pattern, with no underweight participants, 8 participants (20%) classified as normal weight, 24 participants (60%) as overweight, and 8 participants (20%) as obese, with a mean BMI of 27.60 ± 4.06 kg/m². The p-value for the comparison between the two groups was 0.636, indicating no statistically significant difference (Table 1).

Post-intervention data revealed that Group A's BMI distribution shifted slightly, with 0 participants (0%) classified as underweight, 8 participants (20%) as normal weight, 21 participants (52.5%) as overweight, and 11 participants (27.5%) as obese. The mean BMI for Group A increased to 28.05 ± 3.23 kg/m². In Group B, the BMI distribution was 0 participants (0%) underweight, 8 participants (20%) normal weight, 23 participants (57.5%) overweight, and 9 participants (22.5%) obese, with a mean BMI of 27.80 ± 3.87 kg/m². The p-value for the post-intervention comparison was 0.756, showing no significant difference between the two groups (Table 1).

Fasting blood glucose

Pre-intervention

Before the intervention, FBG levels varied between the two groups. In Group A (placebo), none of the participants had FBG levels below 110 mg/dL; 13 participants (32.5%) had levels ranging from 111 to 140 mg/dL, and 27 participants (67.5%) had levels exceeding 140 mg/dL. The mean FBG for this group was 159.60 ± 21.57 mg/dL. In Group B (zinc supplementation), similarly, no participants had FBG levels below 110 mg/dL; 17 participants (42.5%) had levels between 111 and 140 mg/dL, and 23 participants (57.5%) had levels above 140 mg/dL. The mean FBG in Group B was 152.12 ± 22.23 mg/dL. The comparison between these two groups had a p-value of 0.132, indicating no significant difference in pre-intervention FBG levels.

Post-intervention

Following the intervention, significant changes were observed in FBG levels. In Group A, 1 participant (2.5%) had FBG levels below 110 mg/dL, 13 participants (32.5%) had levels between 111 and 140 mg/dL, and 26 participants (65.0%) had levels above 140 mg/dL. The mean FBG for this group was 150.35 ± 22.88 mg/dL. In contrast, Group B showed a substantial shift, with 1 participant (2.5%) having FBG levels below 110 mg/dL, 30 participants (75.0%) with levels between 111 and 140 mg/dL, and 9 participants (22.5%) with levels above 140 mg/dL. The mean FBG in Group B was significantly lower, at 130.60 ± 14.97 mg/dL. The p-value for the post-intervention comparison was 0.001, indicating that zinc supplementation led to a statistically significant reduction in FBG levels compared to the placebo.

Postprandial blood glucose (PPBG)

Pre-Intervention

Initially, postprandial blood glucose (PPBG) levels were similar across groups. In Group A (placebo), none of the participants had PPBG below 200 mg/dL; 20 participants (50%) had levels between 201 and 300 mg/dL, and 20 participants (50%) had levels between 301 and 400 mg/dL. The mean PPBG for this group was 254.35 ± 44.14 mg/dL. In Group B (zinc supplementation), no participants had PPBG below 200 mg/dL; 23 participants (57.5%) had levels between 201 and 300 mg/dL, and 17 participants (42.5%) had levels between 301 and 400 mg/dL. The mean PPBG in Group B was 257.80 ± 65.27 mg/dL. The p-value of 0.783 suggested no significant difference between the groups before the intervention.

Post-Intervention

After the intervention, notable differences emerged. In Group A, 5 participants (12.5%) had PPBG below 200 mg/dL, 33 participants (82.5%) had levels between 201 and 300 mg/dL, and 2 participants (5.0%) had levels between 301 and 400 mg/dL, with a mean PPBG of 235.80 ± 39.86 mg/dL. Conversely, in Group B, 13 participants (32.5%) had PPBG below 200 mg/dL, 27 participants (67.5%) had levels between 201 and 300 mg/dL, and no participants had levels above 300 mg/dL, resulting in a mean PPBG of 210.27 ± 26.20 mg/dL. The p-value of 0.001 indicated a significant reduction in PPBG levels in the zinc supplementation group compared to the placebo.

HbA1c levels and LDL levels: pre-intervention

HbA1c Levels

**Figure 2:**
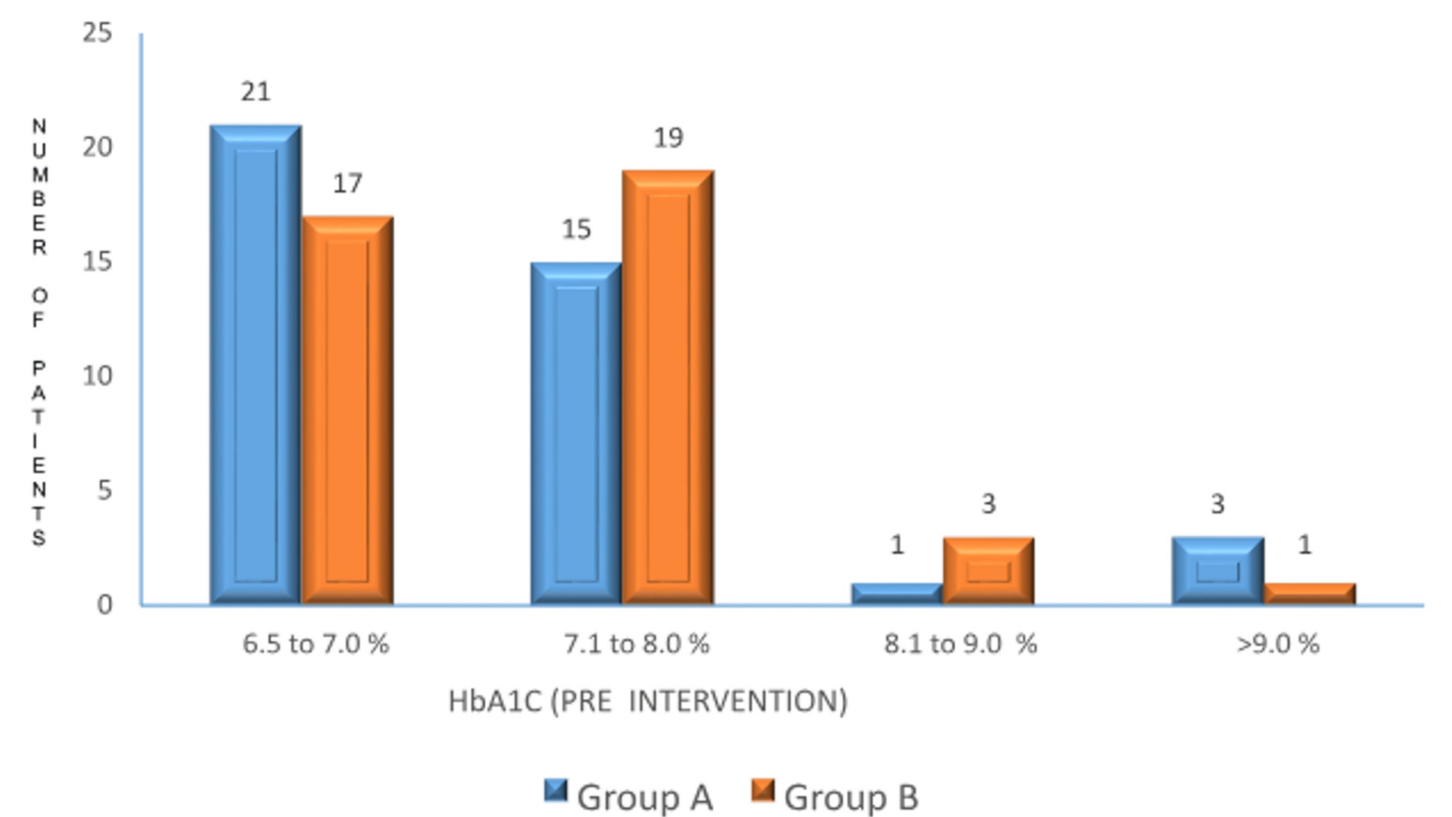
: Pre-intervention HbA1c levels between the two groups p-value: 0.488. No significant difference in HbA1c levels before the intervention. HbA1c: hemoglobin A1c.

Before treatment, there was no significant difference in HbA1c levels between the groups. In Group A (placebo), the mean HbA1c was 7.57 ± 0.69%. In Group B (zinc supplementation), the mean HbA1c was 7.68 ± 0.65%. The p-value was 0.488, indicating no significant difference between the groups (Figure 2).

LDL

**Figure 3:**
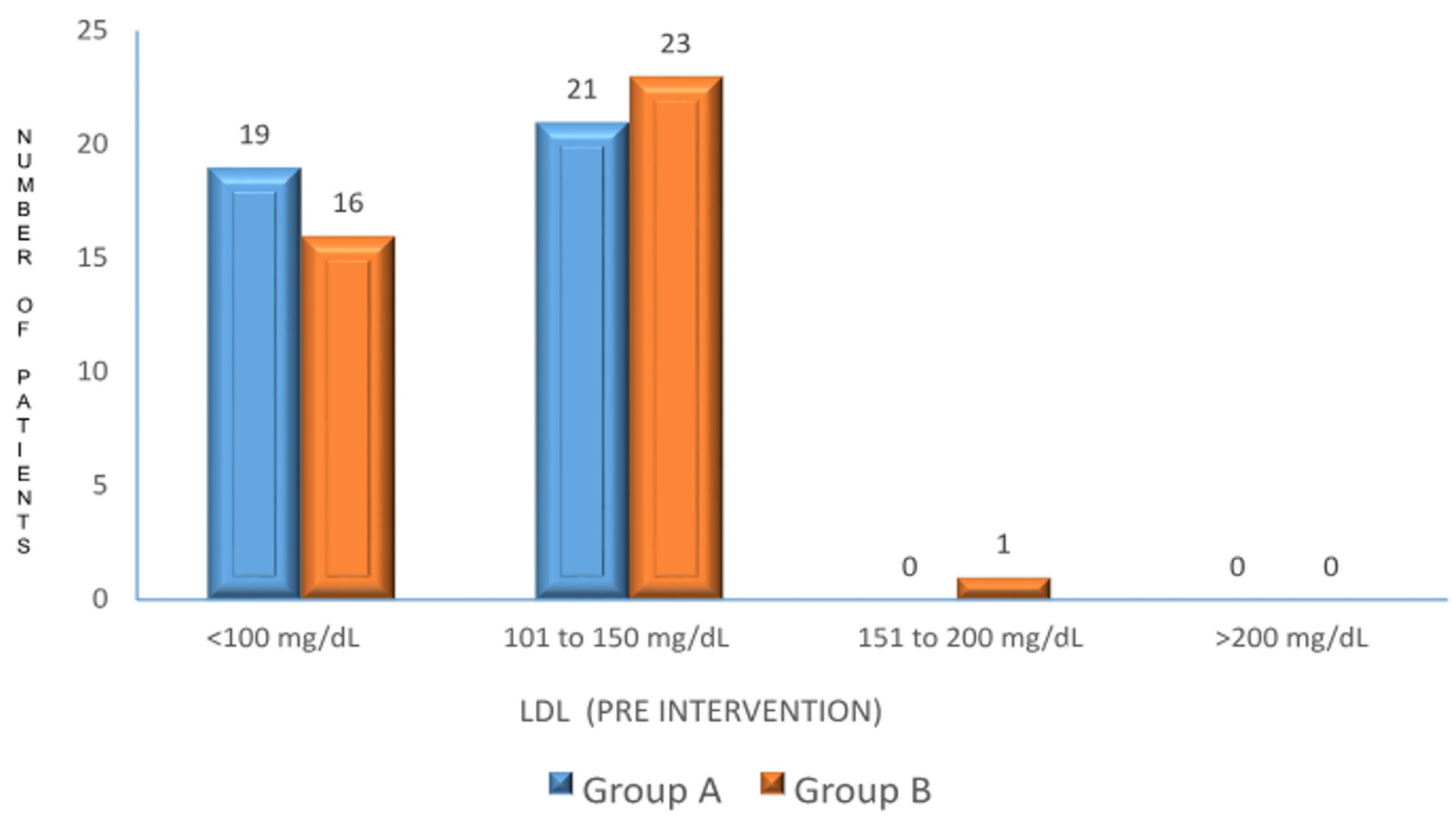
Pre-intervention LDL levels between the two groups p-value: 0.896. No significant difference in LDL levels before the intervention. LDL: low-density lipoprotein.

Prior to the intervention, LDL levels were comparable between the two groups. In Group A, 19 participants (47.5%) had LDL levels below 100 mg/dL, and 21 participants (52.5%) had levels between 101 and 150 mg/dL. In Group B, 16 participants (40%) had LDL levels below 100 mg/dL, and 23 participants (57.5%) had levels between 101 and 150 mg/dL. The p-value was 0.896, indicating no significant difference in LDL levels between the groups (Figure 3).

HbA1c levels and LDL levels: post-intervention

HbA1c

After treatment, Group A had 16 participants (40%) with HbA1c levels between 6.5% and 7.0%, 19 participants (47.5%) with HbA1c levels between 7.1% and 8.0%, and 5 participants (12.5%) with HbA1c levels between 8.1% and 9.0%. The mean HbA1c for Group A decreased to 7.16 ± 0.65%. In contrast, Group B had 22 participants (55%) with HbA1c levels between 6.5% and 7.0%, 18 participants (45%) with HbA1c levels between 7.1% and 8.0%, and no participants with HbA1c levels above 9.0%. The mean HbA1c in Group B was 6.89 ± 0.46%. The p-value of 0.040 indicated a significant reduction in HbA1c levels with zinc supplementation compared to the placebo (Table 3).

LDL

In Group A, 23 participants (57.5%) had LDL levels below 100 mg/dL, and 17 participants (42.5%) had levels between 101 and 150 mg/dL. In Group B, 40 participants (100%) had LDL levels below 100 mg/dL. The p-value of 0.001 indicated a significant reduction in LDL levels with zinc supplementation compared to the placebo (Table 3).

**Table 3:** Post-intervention HbA1c, BMI, LDL, and serum zinc levels between the two groups HbA1c (%): The p-value is 0.040. This value is statistically significant, as it is less than the conventional threshold of 0.05. This indicates a significant difference in HbA1c levels between the two groups. LDL (mg/dL): The p-value is 0.001. This is highly significant, being well below 0.05. Therefore, there is a significant difference in LDL levels between Group A and Group B. BMI: The p-value is 0.756. This is not significant, as it is much greater than 0.05. Thus, there is no significant difference in BMI between the two groups. Serum Zinc (Post): The p-value is 0.003. This is statistically significant, as it is below the threshold of 0.05, suggesting a significant difference in serum zinc levels between the groups.

Measure	Range/category	Group A, n (%)	Group B, n (%)	p-value
HbA1c (%)	6.5 to 7.0	16 (40.00)	22 (55.00)	
	7.1 to 8.0	19 (47.50)	18 (45.00)	
	8.1 to 9.0	5 (12.50)	0 (0.00)	
	>9.0	0 (0.00)	0 (0.00)	
	Total	40 (100.00)	40 (100.00)	
	Mean HbA1c (%)	7.16 ± 0.65	6.89 ± 0.46	0.040
LDL (mg/dL)	<100	23 (57.50)	40 (100.00)	
	101 to 150	17 (42.50)	0 (0.00)	
	151 to 200	0 (0.00)	0 (0.00)	
	>200	0 (0.00)	0 (0.00)	
	Total	40 (100.00)	40 (100.00)	
	p-value			0.001
BMI	<18.5 (Underweight)	0 (0.00)	0 (0.00)	
	18.5-24.9 (Normal weight)	8 (20.00)	8 (20.00)	
	25.0-29.9 (Overweight)	21 (52.50)	23 (57.50)	
	>30.0 (Obese)	11 (27.50)	9 (22.50)	
	Total	40 (100.00)	40 (100.00)	
	Mean BMI (kg/m²)	28.05 ± 3.23	27.80 ± 3.87	0.756
Serum zinc	<50	1 (2.50)	0 (0.00)	
	51 to 74.0	29 (72.50)	14 (35.00)	
	75.0 to 100.0	9 (22.50)	26 (65.00)	
	>100.0	1 (2.50)	0 (0.00)	
	Total	40 (100.00)	40 (100.00)	
	Mean zinc (µg/dL)	70.12 ± 12.38	77.22 ± 7.40	0.003

Triglycerides

Triglyceride levels were similar before the intervention, with no significant difference (p-value = 0.564). After treatment, Group A had a mean TG of 111.30 ± 38.46 mg/dL, while Group B had a significantly lower mean TG of 91.70 ± 12.75 mg/dL. The p-value of 0.003 highlighted a significant reduction in TG levels with zinc supplementation.

High-density lipoprotein

HDL levels were similar pre-intervention, with no significant difference (p-value = 0.752). Post-intervention, Group A’s mean HDL increased to 49.04 ± 7.18 mg/dL, whereas Group B’s mean HDL increased to 52.73 ± 6.84 mg/dL. The p-value of 0.204 suggested no significant difference between the groups.

Total cholesterol

Pre-intervention

Before the intervention, total cholesterol levels were similar (p-value = 0.659).

Post-intervention

Group A’s mean cholesterol decreased to 173.92 ± 23.23 mg/dL, while Group B’s mean cholesterol was lower at 160.20 ± 25.69 mg/dL. The p-value of 0.014 indicated a significant reduction in cholesterol levels with zinc supplementation.

Very-low-density lipoprotein

VLDL levels showed no significant difference before the intervention (p-value = 0.444). Post-intervention, Group A’s mean VLDL slightly decreased to 27.25 ± 9.42 mg/dL, while Group B’s mean VLDL decreased to 24.65 ± 9.00 mg/dL. The p-value of 0.211 suggested no significant difference.

Erythrocyte sedimentation rate

Pre-intervention

There was no significant difference in the mean ESR levels between the two groups before treatment. Group A had a mean ESR of 27.30 ± 15.64 mm/hour. Specifically, 10 participants (25%) in Group A had ESR levels below 20 mm/hour, 18 participants (45%) had levels between 20 and 30 mm/hour, and 12 participants (30%) had levels above 30 mm/hour. In Group B, the mean ESR was 23.67 ± 9.60 mm/hour, with 14 participants (35%) having ESR levels below 20 mm/hour, 20 participants (50%) between 20 and 30 mm/hour, and 6 participants (15%) above 30 mm/hour. The p-value of 0.215 indicated no significant difference in ESR levels between the groups before treatment.

Post-intervention

After treatment, Group A had a mean ESR of 26.27 ± 15.75 mm/hour. In this group, 12 participants (30%) had ESR levels below 20 mm/hour, 16 participants (40%) had levels between 20 and 30 mm/hour, and 12 participants (30%) had levels above 30 mm/hour. Group B had a mean ESR of 23.15 ± 8.56 mm/hour, with 18 participants (45%) having ESR levels below 20 mm/hour, 16 participants (40%) between 20 and 30 mm/hour, and 6 participants (15%) above 30 mm/hour. The p-value of 0.211 indicated no significant difference in ESR levels between the groups post-intervention.

Serum zinc levels

Pre-intervention

Before the intervention, the mean serum zinc level for Group A (placebo) was 70.17 ± 14.25 µg/dL. In this group, 15 participants (37.5%) had serum zinc levels below 60 µg/dL, 20 participants (50%) had levels between 60 and 80 µg/dL, and 5 participants (12.5%) had levels above 80 µg/dL. Group B (zinc supplementation) had a mean serum zinc level of 67.32 ± 8.11 µg/dL, with 18 participants (45%) having levels below 60 µg/dL, 20 participants (50%) between 60 and 80 µg/dL, and 2 participants (5%) above 80 µg/dL. The p-value of 0.275 indicated no significant difference in serum zinc levels between the two groups before the intervention.

Post-intervention

After the intervention, Group A’s mean serum zinc level was 70.12 ± 12.38 µg/dL. In this group, 12 participants (30%) had serum zinc levels below 60 µg/dL, 22 participants (55%) had levels between 60 and 80 µg/dL, and 6 participants (15%) had levels above 80 µg/dL. In contrast, Group B’s mean serum zinc level was significantly higher at 77.22 ± 7.40 µg/dL, with 5 participants (12.5%) having levels below 60 µg/dL, 20 participants (50%) between 60 and 80 µg/dL, and 15 participants (37.5%) above 80 µg/dL. The p-value of 0.003 highlighted a statistically significant difference between the two groups.

## Discussion

This study aimed to evaluate the impact of zinc supplementation on glycemic control and lipid profiles in newly diagnosed T2DM patients. Our findings demonstrate that zinc supplementation significantly improved FBG, postprandial blood glucose (PPBG), and hemoglobin A1c (HbA1c) levels, as well as positively influenced lipid profiles, compared to placebo. Zinc deficiency is well-documented in its association with diabetes development, potentially delaying the onset of diabetes from prediabetes. Zinc plays a critical role in insulin secretion and is closely linked with pancreatic β cells, which are essential for insulin production. This connection underlines zinc's potential role in managing both Type 1 and Type 2 DM. Prior studies have shown that zinc supplementation can enhance insulin sensitivity and secretion in individuals with IGT, although excessive intake may have adverse effects. In India, where demographic factors such as a higher proportion of lean diabetes patients are prevalent, community-based surveys are necessary to assess zinc deficiency across rural and urban populations. Understanding zinc’s clinical effectiveness in managing Type 2 diabetes could provide a cost-effective public health intervention for diabetes management in this context.

Participants in this study had a mean age of 48.55 ± 8.67 years in the placebo group and 47.62 ± 7.49 years in the zinc group, with no significant difference in age (p = 0.611). The male-to-female ratio was comparable between groups, with 0.9 in the zinc group and 0.7 in the placebo group, aligning with WHO data, indicating that the groups were well-matched for these demographic characteristics. The BMI of participants showed no significant change following zinc supplementation (pre-intervention: 27.60 kg/m²; post-intervention: 27.80 kg/m², p = 0.756). This stability is consistent with the findings of Abdollahi et al. [12], who reported similar results. The lack of significant change in BMI suggests that zinc supplementation primarily affects glucose and lipid metabolism rather than body weight. Thus, the above parameters show that both body weight and gender distribution do not impact the results of our study.

Zinc supplementation led to a significant reduction in FBG by 21.52 mg/dL compared to a 9.25 mg/dL reduction with placebo (p = 0.001). This finding highlights the effectiveness of zinc in improving fasting glucose levels in newly diagnosed T2DM patients. Our results are consistent with previous studies by Jayawaradhane et al. [13] and Gunasekara et al. [14], who also reported significant reductions in FBG with zinc supplementation. Post-intervention, zinc supplementation also reduced PPBG by 47.53 mg/dL, compared to an 18.55 mg/dL reduction with placebo (p = 0.001). This significant difference underscores zinc's role in managing postprandial glucose levels, although data on this effect are limited. Additionally, there was a substantial decrease in HbA1c levels by 0.79% compared to a 0.41% decrease with placebo (p = 0.04). This reduction aligns with findings from Jafarnejad et al. [15], demonstrating the HbA1c-lowering benefits of zinc. The significant improvement in HbA1c suggests that zinc supplementation can effectively enhance long-term glycemic control. Thus, the study proves that zinc supplementation in newly diagnosed Type 2 diabetic patients results in significant improvement in glycemic parameters.

Our study observed reductions in LDL cholesterol, triglycerides (TG), and total cholesterol (TC) with zinc supplementation, consistent with results from Asbaghi et al. [16]. Hence, zinc supplementation resulting in a lipid-lowering effect leads to better microvascular and macrovascular outcomes. Although HDL cholesterol levels increased, this change was not statistically significant, and there was no significant effect on VLDL cholesterol. These findings support the role of zinc in improving lipid profiles in T2DM patients.

No significant changes in ESR were observed with zinc supplementation. This result aligns with other studies, which also reported no meaningful changes in ESR and other indicators such as renal function tests and hemograms. Prior to supplementation, most participants had serum zinc levels below 75 µg/dL. Post-intervention, zinc supplementation significantly increased serum zinc levels in Group B (p = 0.003). This finding corroborates studies by Sen et al. [11], who reported lower serum zinc levels in diabetic patients. The significant increase in serum zinc levels highlights the efficacy of zinc supplementation in correcting zinc deficiency.

The studies done on zinc supplementation are very few in number; hence, longer studies with larger populations are required to fully understand the benefits of zinc supplementation on glycemic control.

Limitations

The study faced a few limitations that impacted the generalizability and interpretation of its findings. First, the sample size was too small to extrapolate results to the general population, and the short follow-up period limited the assessment of long-term effects. Additionally, ethical considerations prevented a full evaluation of zinc’s efficacy as a monotherapy. There were also variations in baseline parameters, such as serum zinc status, blood glucose, and lipid levels, which could have influenced the outcomes. Furthermore, differences in zinc doses, formulations, sample sizes, and study durations across different studies contributed to inconsistent results. Lastly, the limited availability of data on zinc intake from other sources, such as diet, made it challenging to account for all variables that might affect the study’s findings.

## Conclusions

When compared to a placebo group, zinc supplementation improves glycemic control and lipid profiles in newly diagnosed Type 2 diabetic patients. It appears, therefore, that the beneficial effects of zinc supplementation on metabolic parameters are primarily observed in zinc-deficient individuals or in conditions that cause zinc deficiency, such as diabetes.

In comparison to the placebo group, metformin and zinc supplementation demonstrated superior glycemic control in our trial, which involved individuals with recently diagnosed Type 2 diabetes. After one year, HbA1c, FBS, and PPBG were significantly improved in the zinc-supplemented group compared to the placebo group. Hence, we can conclude that zinc supplementation with metformin can be beneficial for T2DM patients.

## References

[1] Lakhtakia R (2013). The history of diabetes mellitus. Sultan Qaboos Univ Med J.

[2] MacCracken J, Hoel D (1997). From ants to analogues. Puzzles and promises in diabetes management. Postgrad Med.

[3] Elsayed S, Soliman AT, De Sanctis V, Fawzy D, Ahmed S, Alaaraj N (2023). Insulin-induced lipodystrophy and predisposing factors in children and adolescents with type 1 diabetes mellitus (T1DM) in a tertiary care Egyptian center. Acta Biomed.

[4] Ranasinghe P, Pigera S, Galappatthy P, Katulanda P, Constantine GR (2015). Zinc and diabetes mellitus: understanding molecular mechanisms and clinical implications. Daru.

[5] Bjørklund G, Dadar M, Pivina L, Doşa MD, Semenova Y, Aaseth J (2020). The role of zinc and zopper in Insulin resistance and diabetes mellitus. Curr Med Chem.

[6] Hasanato RM (2020). Trace elements in type 2 diabetes mellitus and their association with glycemic control. Afr Health Sci.

[7] Viktorínová A, Toserová E, Krizko M, Duracková Z (2009). Altered metabolism of copper, zinc, and magnesium is associated with increased levels of glycated hemoglobin in patients with diabetes mellitus. Metabolism.

[8] Ekin S, Mert N, Gunduz H, Meral I (2003). Serum sialic acid levels and selected mineral status in patients with type 2 diabetes mellitus. Biol Trace Elem Res.

[9] Darenskaya MA, Kolesnikova LI, Kolesnikov SI (2021). Oxidative stress: pathogenetic role in diabetes mellitus and its complications and therapeutic approaches to correction. Bull Exp Biol Med.

[10] Ma YM, Tao RY, Liu Q (2011). PTP1B inhibitor improves both insulin resistance and lipid abnormalities in vivo and in vitro. Mol Cell Biochem.

[11] Yoshikawa Y, Ueda E, Kojima Y, Sakurai H (2004). The action mechanism of zinc(II) complexes with insulinomimetic activity in rat adipocytes. Life Sci.

[12] Abdollahi S, Toupchian O, Jayedi A, Meyre D, Tam V, Soltani S (2020). Zinc supplementation and body weight: a systematic review and dose-response meta-analysis of randomized controlled trials. Adv Nutr.

[13] Jayawardena R, Ranasinghe P, Galappatthy P, Malkanthi R, Constantine G, Katulanda P (2012). Effects of zinc supplementation on diabetes mellitus: a systematic review and meta-analysis. Diabetol Metab Syndr.

[14] Gunasekara P, Hettiarachchi M, Liyanage C, Lekamwasam S (2011). Effects of zinc and multimineral vitamin supplementation on glycemic and lipid control in adult diabetes. Diabetes Metab Syndr Obes.

[15] Jafarnejad S, Saremi S, Jafarnejad F, Arab A (2016). Effects of a multispecies probiotic mixture on glycemic control and inflammatory status in women with gestational diabetes: a randomized controlled clinical trial. J Nutr Metab.

[16] Asbaghi O, Fatemeh N, Mahnaz RK (2020). Effects of chromium supplementation on glycemic control in patients with type 2 diabetes: a systematic review and meta-analysis of randomized controlled trials. Pharmacol Res.

